# Engineering proteinase K using machine learning and synthetic genes

**DOI:** 10.1186/1472-6750-7-16

**Published:** 2007-03-26

**Authors:** Jun Liao, Manfred K Warmuth, Sridhar Govindarajan, Jon E Ness, Rebecca P Wang, Claes Gustafsson, Jeremy Minshull

**Affiliations:** 1 Department of Computer Science, University of California, Santa Cruz, CA 95064 USA; 2 DNA 2.0, 1430 O'Brien Drive, Suite E, Menlo Park, CA 94025, USA

## Abstract

**Background:**

Altering a protein's function by changing its sequence allows natural proteins to be converted into useful molecular tools. Current protein engineering methods are limited by a lack of high throughput physical or computational tests that can accurately predict protein activity under conditions relevant to its final application. Here we describe a new synthetic biology approach to protein engineering that avoids these limitations by combining high throughput gene synthesis with machine learning-based design algorithms.

**Results:**

We selected 24 amino acid substitutions to make in proteinase K from alignments of homologous sequences. We then designed and synthesized 59 specific proteinase K variants containing different combinations of the selected substitutions. The 59 variants were tested for their ability to hydrolyze a tetrapeptide substrate after the enzyme was first heated to 68°C for 5 minutes. Sequence and activity data was analyzed using machine learning algorithms. This analysis was used to design a new set of variants predicted to have increased activity over the training set, that were then synthesized and tested. By performing two cycles of machine learning analysis and variant design we obtained 20-fold improved proteinase K variants while only testing a total of 95 variant enzymes.

**Conclusion:**

The number of protein variants that must be tested to obtain significant functional improvements determines the type of tests that can be performed. Protein engineers wishing to modify the property of a protein to shrink tumours or catalyze chemical reactions under industrial conditions have until now been forced to accept high throughput surrogate screens to measure protein properties that they hope will correlate with the functionalities that they intend to modify. By reducing the number of variants that must be tested to fewer than 100, machine learning algorithms make it possible to use more complex and expensive tests so that only protein properties that are directly relevant to the desired application need to be measured. Protein design algorithms that only require the testing of a small number of variants represent a significant step towards a generic, resource-optimized protein engineering process.

## Background

Protein properties that are relevant to real-world applications are often difficult to manipulate using either of the current protein engineering paradigms [1-3]: structure-based protein design [4,5] or directed evolution [6-8]. Both methods have shortcomings and advantages that have been discussed and compared elsewhere [1-3]. Chief amongst the limitations of both methods is the requirement for high throughput computational or physical tests to evaluate protein variants for suitability to a specific application. A common problem with both approaches is that frequently there are no high throughput tests for real applications. For example, there are no high throughput tests for measuring how well a protease will remove grass stains from jeans, how quickly an antibody will shrink a tumour, or how immunogenic a potential vaccine antigen will be. As a consequence, protein engineers are frequently forced to compromise. Thus a structure-based approach in which the effects of large numbers of amino acid changes on the active site are calculated may require the protein engineer to consider only the affinity of an enzyme for its substrate and product while ignoring the effects that temperature and solvent conditions may have on the enzyme. Similarly an empirical library based approach in which large numbers of randomly produced viral antigen variants are tested for activity may allow the protein engineer to measure their binding to antibodies already known to be neutralizing, but would prohibit direct measurement of the production of such antibodies in animals exposed to the antigens.

Many non-biotechnological engineering endeavours pose similar challenges to those found in protein engineering: a large number of independent variables and cost-prohibitions against exhaustive search. Such diverse tasks as fuel formulation, clinical trial design and chemical process optimization are solved using experimental designs to combine variables in specific ways, and regression analysis techniques to dissect out the contribution of each variable to the outcome [9]. The common goal in all these areas of optimization is to keep the total number of activity measurements small enough to allow complex functional tests that are directly relevant to the final application.

Multivariate data analysis has been used to optimize small molecules and peptides for nearly a quarter of a century [10-16]. In their paper describing chemical synthesis of a gene in 1984, Benner and colleagues suggested that systematic variation of amino acids could provide an understanding of the relationship between a protein's sequence and its function [17]. Until recently, however, synthesis of specifically designed individual genes has been sufficiently difficult to effectively preclude the construction of designed gene sets and meaningful testing of analytical predictions. Such efforts have thus been largely confined to the synthesis of very small numbers of discrete polynucleotide [18] or protein variants [19], or to the analysis of variants produced in a library [20-22].

A synthetic biology approach to protein engineering has been enabled by recent advances in gene synthesis technology [23-26] that permit cost-effective synthesis of individually specified gene sequences instead of relying on creation of libraries of variant sequences [27,28]. The feasibility of producing tens or hundreds of protein variants in which all amino acid changes are precisely specified allows the sequences and activities of these variants to be analyzed using multivariate regression and machine learning techniques adapted from optimization tasks found in other engineering disciplines.

We have tested this protein engineering approach by increasing the activity and heat stability of proteinase K. We selected 24 amino acid substitutions, then designed, synthesized and tested 59 genes containing combinations of these changes. We tested 8 different machine learning algorithms for their ability to identify the amino acid changes with a beneficial effect on proteinase K activity by using them to design new variants with improved combinations of substitutions. In 3 design cycles we synthesized genes encoding a total of 95 enzymes (~100 kb of synthetic genes), some of which had 20 times higher activity than the wild type protein. All 8 algorithms produced enzyme designs that were substantially improved over wild type. The results show that machine learning models of protein sequence and activity combined with efficient gene synthesis can be valuable tools in engineering proteins with improved properties.

## Results and Discussion

### 1. Selection of proteinase K as a test system

To test machine learning-based protein engineering we chose to optimize proteinase K-catalyzed hydrolysis of the tetrapeptide N-Succinyl-Ala-Ala-Pro-Leu *p*-nitroanilide following a heat-treatment of the enzyme. We selected this activity because it mimics a key characteristic of practical protein optimization; target activities frequently result from a combination of protein properties, in this case expression and post-translational processing in a heterologous host, catalytic activity and thermostability.

The gene encoding proteinase K from *Tritirachium album *[29] was re-synthesized with an *E. coli *codon bias [30,31] and cloned into an arabinose-inducible *E. coli *expression vector. The nucleotide and amino acid sequences of this initial ("wild-type") proteinase K sequence are shown in Additional file 1.

### 2. Engineering proteinase K: design methods

#### 2.1 Overview of the method

The protein engineering method described here involves the following steps.

i) Selection of amino acid substitutions to incorporate into a target protein.

ii) Design of protein variants containing different combinations of those substitutions.

iii) Synthesis of genes encoding the protein variants.

iv) Expression of the protein variants.

v) Measuring the activity of the protein variants.

vi) Analysis of protein variant sequences and activities to assess the contribution of each amino acid substitution.

vii) Design of a new set of variants using the information from vi).

viii) Iteration of steps iii) to vii).

**Figure 1:**
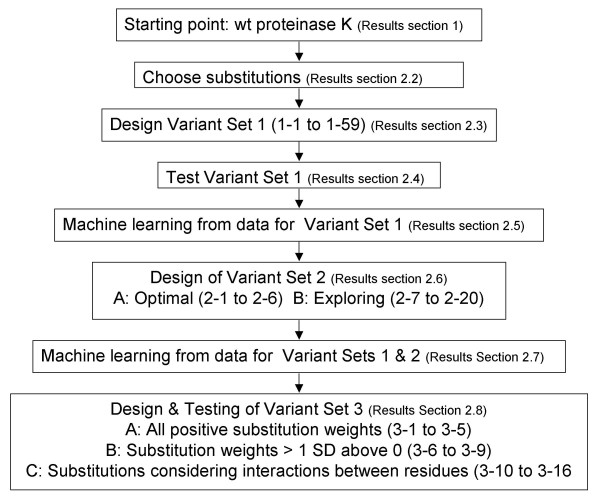
**Flowchart of protein engineering design and testing process**. The figure shows an overview of the experimental flow described in this work. Details are provided for each step in the indicated section of Results and Discussion.

These steps are described in detail for the engineering of proteinase K in the Results sections noted in Figure 1.

#### 2.2 Selection of amino acid substitutions

We planned on synthesizing a total of less than 100 variants containing combinations of a limited set of amino acid substitutions. To define a search space that could be effectively explored by synthesizing such a small number of variants we chose to use twenty four amino acid substitutions within the ~370 amino acid proteinase K: less than 0.5% of the total number of single amino acid changes possible.

To select the substitutions, a set of serine proteases with >30% amino acid identity to proteinase K were identified by using the BLAST algorithm to search Genbank. This search produced 3 groups of homologous sequences. Group A contained the wild type and 5 close homologs (>90% amino acid identity). Group B contained 42 more distant homologs (between 30% and 90% amino acid identity). Group C contained 11 homologs (>30% amino acid identity) that were either reported in the literature to be thermostable or were >90% identical to a known thermostable sequence. Genbank accession numbers are provided in Additional file 1.

The homologs were aligned using clustalW[32], to identify the amino acids in each homolog that corresponded with the amino acid found in wild type proteinase K at each position. To increase the probability that at least some of the substitutions would increase activity, we selected 24 substitutions based on several different criteria [33]: (i) the substitution was reported in the literature to increase the stability of the serine protease subtilisin (N95C, P97S, E138A, M145F, L299C, I310K) [34,35]; (ii) the amino acid occurred within the homolog set (S107D); (iii) the amino acid occurred within >70% of homologs from thermophilic organisms and within other homologs (S123A, I132V, L180I, R237N, S273T, G293A, K332R, S337N); (iv) the amino acid was found within the homolog set and the substitution from the wild type residue is favourable in the Dayhoff substitution matrix (V167I, A199S, V267I) [36]; (v) principal component analysis of amino acids responsible for clustering of homologs from thermophilic organisms (K208H, A236V) [37]; (vi) literature reports that the P5S substitution has a stabilizing effect in subtilisin [34,35] AND appearance of a P to S substitution in the closest proteinase K homolog (P265S, P355S); (vii) the change occurred in a close homolog that is also thermostable (Y151A) and (viii) a random mutation identified during synthesis of the wild type (Y194S). This information is summarized in Table 1

We emphasize that the method used for choosing amino acid substitutions is independent of the subsequent machine learning analysis. Substitutions could be selected by any of the many available methods including analysis of protein structures [38] or comparison of homologous sequences [39]. A more detailed review of combined methods for substitution selection has been published elsewhere[40].

#### 2.3 Design of variant set 1

In order to test the machine learning algorithms, we needed to obtain a set of variants with corresponding activity measurements. For the most accurate analysis each substitution should be approximately equally represented, and should occur with as many different substitutions (ie in different sequence contexts) as possible. We encountered two somewhat related obstacles to creating such a variant set. Firstly, all of the substitutions were previously untested, so we did not know how many would completely inactivate the enzyme. Secondly we did not know how tolerant proteinase K would be to changes, that is how many amino acids we could change in a single variant and retain activity. The initial set of 59 variants was therefore designed in several stages as we obtained information about these parameters. The sequences of all variants synthesized are shown in Additional file 2.

i) First a set of 24 variants was designed with combinations of substitutions selected randomly but with the constraint that each of the 24 substitutions occurred 6 times and each variant contained 6 substitutions (1–2 to 1–25, design method B in Additional file 2). Of these 24 variants only one (1–13) was active after heat-treatment. To determine whether the low survival rate was because the substitutions destroyed all proteinase K activity, or because the substitutions primarily affected the heat sensitivity, we also measured the activity of all 24 variants without heating. Under these less stringent conditions we found three additional variants that were active (1–2, 1–6 and 1–12). Eighteen of the 24 substitutions were present in 1 or more of these 4 variants with detectable proteinase K activity and thus did not completely inactivate the enzyme.

ii) To see which of the remaining 6 substitutions destroyed proteinase K activity, we synthesized variants containing the substitutions that had not occurred within an active variant (1–26 to 1–33, design method C in Additional file 2). Variants containing N95C, P97S, E138A, A236V and L299C were completely inactive, so these substitutions were eliminated from further designs.

iii) To obtain a larger number of active variants for modeling using machine learning we designed 10 variants by arbitrarily combining 3 substitutions that had appeared previously in active variants (1–34 to 1–43, design method D in Additional file 2) and 6 variants by arbitrarily combining 5 substitutions that had appeared previously in active variants (1–44 to 1–49, design method E in Additional file 2).

iv) Finally we performed a manual analysis of the activity data from the first 49 variants, combining substitutions that occurred frequently within active variants (1–50 to 1–59, design method F in Additional file 2).

#### 2.4 Testing variant set 1

**Figure 2:**
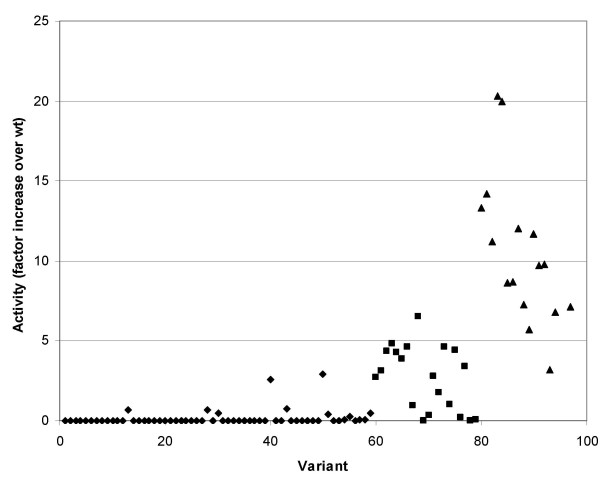
**Three cycles of proteinase K variant design and testing**. Mean activity measurements of the 3 sets of proteinase K variants are shown. Set 1 (diamonds) is the initial set of 59 variants. Set 2 (squares, 20 variants) was designed using the activities of Set 1. Set 3 (triangles, 16 variants) was designed based on sets 1 and 2. Activities towards N-Succinyl-Ala-Ala-Pro-Leu p-nitroanilide were measured at 37°C following a 5 minutes heat treatment of the enzyme at 68°C. Activities are expressed relative to the mean activity of 2 replicates of the wild-type proteinase K.

To associate protein sequences with functions, we tested the ability of the proteinase K variants to hydrolyze N-Succinyl-Ala-Ala-Pro-Leu *p*-nitroanilide. Proteinase K variants were expressed in *E. coli *and purified over a Ni-NTA column. The purified proteins were heated to 68°C for 5 minutes, cooled and diluted into reaction buffer containing substrate. The activities measured are shown in Figure 2 and in Additional file 2, which also show the activities of variants designed in two subsequent design cycles.

Only 19 of the 59 enzymes in variant set 1 had detectable activities after heat treatment [see Additional file 2]. As described in section 2.5, we wished to analyze this dataset using machine learning algorithms to calculate the values of 20 parameters. Using a dataset this sparse will cause inaccuracies for the machine learning algorithms. To increase the number of datapoints without increasing the number of sequences synthesized we also measured the activities of all of the enzymes in variant set 1 without the heating step. We reasoned that this would provide additional information, differentiating combinations of substitutions that eliminated enzyme activity entirely from those which were simply unable to confer thermostability. More than half (32 of 59) of the variants in set 1 were active without a heating step (Additional file 2).

#### 2.5 Choice of machine learning algorithms and analysis of variant set 1

We wished to learn which amino acid substitutions increased activity and which were detrimental by analyzing the sequences and activities of the proteinase K variants. The initial dataset was rather sparse: only two variants in the initial set (1–40 and 1–50) had an activity exceeding that of the wild type, by 2.8-fold and 3.3-fold respectively, while 45 possessed less than 10% of the wild type activity. Despite the generally low activities of the first set of variants, there was a range of activities that we analyzed by machine learning.

To do this we first eliminated five substitutions (N95C, P97S, E138A, A236V and L299C) because variants with any of these substitutions did not have any detectable activity (Additional file 2). We then considered the reduced set of 19 substitutions, representing each variant as a 19 dimensional bit vector x_i_, where x_i,j _is 1 if there is a substitution in the variant at position j. We used a bit vector since only one possible amino acid substitution was used at each position. A test of a protein variant was encoded as a pair (x_i_, y_i_), where x_i _represents the variant and y_i _the activity measured for this variant.

We selected 8 different machine learning algorithms to analyze the data. We used 8 different algorithms because we had no way of knowing which, if any, would be suitable for analyzing protein sequences and activities. The algorithms differ in two main ways. First in the way in which they calculate the differences between the measured activity and the predicted activity (the "loss"), for example whether they use the square of the differences between measured and predicted activities (square loss), or whether they place more weight on differences between measured and predicted activities for the more active variants (matching loss). Second, the algorithms use different regularization functions, which determine for example whether preferred solutions use many small weights (2-norm) or fewer large weights (1-norm). The algorithms used were: ridge regression (RR) [41]; least absolute shrinkage and selection operator (Lasso) [42]); partial least square regression (PLSR) [43]; support vector machine regression (SVMR) [44]; linear programming support vector machine regression (LPSVMR) [45]; linear programming boosting regression (LPBoostR) [46]; matching loss regression (MR) [47,48]; one-norm regularization matching-loss regression (ORMR) [47,48]. See Additional file 1 for detailed descriptions of the algorithms.

Each algorithm was used to build linear models of the sequence and activity by calculating a 20-dimensional weight vector **w**, where the activity of a variant x_j _is estimated as y˜
 MathType@MTEF@5@5@+=feaafiart1ev1aaatCvAUfKttLearuWrP9MDH5MBPbIqV92AaeXatLxBI9gBaebbnrfifHhDYfgasaacH8akY=wiFfYdH8Gipec8Eeeu0xXdbba9frFj0=OqFfea0dXdd9vqai=hGuQ8kuc9pgc9s8qqaq=dirpe0xb9q8qiLsFr0=vr0=vr0dc8meaabaqaciaacaGaaeqabaqabeGadaaakeaaieaacuWF5bqEgaacaaaa@2E3B@_i _= (∑_j = 1..19_w_j_x_i,j_) + w_20_. The weight w_j _is associated with the j-th substitution, providing a measure of the effect of the j-th substitution on proteinase K activity. The last weight w_20 _is an additive shift. The machine learning algorithms were used to select values for w_j _that resulted in the best correlation between the activities that had been measured for each variant and the activities predicted by the weight vectors **w**. To do this we created 1000 subsamples of the training set (the set of all (x_i_, y_i_) pairs used for a cycle of machine learning) by leaving out 5 randomly chosen variant sequences for each such subsample. For all 8 algorithms we calculated the mean value and the standard deviation of each substitution weight w_j _over the 1000 subsamples of the training set.

#### 2.6 Design of variant set 2

One objective in designing a second variant set was to see whether variants based on the results of machine learning analysis had improved activity relative to the training set. We also wished to obtain additional data so that we could perform a second round of machine learning-based variant design, should the first round prove successful. Variant set 2 was therefore designed in 3 parts.

Initially we used each machine learning algorithm to select the sequence that it predicted would have the highest activity using the heated activity data from the first set (variants 2-1 to 2–6, design method G in Additional file 2). The effect of any substitution may depend upon the other substitutions with which it occurs (see Section 3.7). The fewer times that a substitution has been seen, the less accurately its average effect is known. We reasoned that a substitution should be seen at least three times to estimate a meaningful average effect (if the effect in one context is positive, and in another context is negative, a third context will provide a "tiebreaker"). We therefore excluded substitutions that had occurred in active variants fewer than 3 times. To further reduce the chances of incorporating an apparently positive substitution that actually had a negative effect we only included those substitutions whose weights exceeded a threshold. We first normalized all our activities against the wild type, resulting in activity 1 for the wild type. We then chose the threshold as 0.04 = 1/25, where 25 is the original number of weights (24 substitutions plus a bias term). Note that the final number of substitutions somewhat smaller (19). This led to the exclusion of M145F, S123A, E132A and V267I from variants 2-1 to 2–6. In designing variant set 3, we improved our design method, using the standard deviation for each substitution weight instead of an arbitrary threshold (see Section 2.8).

We designed a further 14 variants in set 2 to more thoroughly explore the search space close to already tested variants and thus to provide sufficient data for a further cycle of machine learning. Six of these (2–7 to 2–12, design method H in Additional file 2) were designed using each machine learning algorithm in turn to select the variant with the largest predicted activity based on the mean weight for each substitution. To ensure that we designed sequences that were different from the first six, we only allowed variants between 3 and 5 amino acid changes from any tested variant of set 1 or any variant already chosen for inclusion in set 2. The lower bound of distance 3 assured that new and significantly different variants were chosen, and the upper bound of distance 5 limited the risk of encountering non-viable combinations.

The last 8 variants of set 2 (2–13 to 2–20, design method I in Additional file 2) were designed in the same way as 2–7 to 2–12, except that instead of using the activities after heating for the machine learning, we used the activities before heating. The reason for this was that only 19 of the 59 variants had detectable activities after heating, but 32 had detectable activities before heating. The unheated measurements thus provided a better dataset for machine learning and we reasoned that they would increase the diversity of designs of active proteinase K variants. This is discussed in more detail in section 3.6. The 0.04 threshold was not subtracted from the weights for designs H and I (our aim was to design a set of active but different variants) and the four substitutions that were excluded from 2-1 through 2–6 (M145F, S123A, E132A and V267I) were included in 2–7 through 2–20.

#### 2.7 Testing and machine learning analysis of variant set 2

The first set of machine learning-based designs significantly outperformed those based on a manual "expert" analysis in set 1. Thirteen of the twenty variants in set 2 were more active than wild-type proteinase K, with 8 more active than the most active variant from set 1 (Figure 2 and Additional file 2]). Encouraged by this result we performed a second cycle of machine learning.

**Figure 3:**
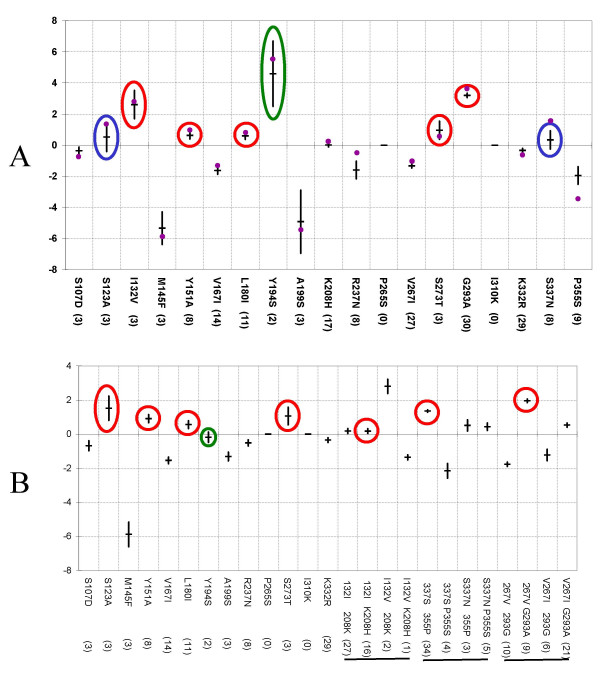
**Substitution weight mean and standard deviation values produced by the MR algorithm**. We created 1000 subsamples of the training set (the sequences and non-zero activities of variants from sets 1 and 2) by leaving out 5 randomly selected variants from each subsample. A: The MR (matching loss) algorithm was used to calculate substitution weights for each subsample. The mean values from the 1000 subsamples are indicated by horizontal notches. Error bars represent one standard deviation of the 1000 calculated substitution weights. Substitutions are indicated below the graph with the number of occurrences in the training set in parentheses. Each substitution is described by a single weight. Variant 3–4 was designed to include all substitutions with positive mean weight that occur at least 3 times in the training set (red and blue circles). Note that substitution Y194S (green circle) was not selected since it occurred less than 3 times in the training set. Variant 3–9 included all substitutions that occurred at least 3 times and whose mean weight was at least one standard deviation above zero (red circles only). Substitution weights calculated from the entire dataset instead of the mean of 1000 subsamples are shown as purple circles. B: The MR algorithm was used to calculate substitution weights as in A, except that models were tested by expanding each pair in turn into 4 terms and selecting the pair that most improved the model. In this example each substitution is described by a single weight except for the 3 pairs (132,208), (337,355), (267,293) which are modeled by 4 weights each. Re d circles indicate the substitutions selected to design variant 3–14. Note that substitution combination I132V 208K was not selected since it occurred less than 3 times in the training set.

The sequence and activity data from the first and second set of variants was combined and analyzed as before using each of 8 different machine learning algorithms to build linear models of the sequence and activity as described in section 2.5. The mean weights and standard deviations calculated by each algorithm are shown in Table 2, and shown graphically for one algorithm (MR) in Figure 3A.

Because we now had more sequence and activity data, we also performed a rudimentary test of regression models that consider epistatic interactions between the selected substitutions. We did this by asking whether models containing epistatic interactions would result in a better fit between the observed and predicted activities of the variants in the training set.

Ideally we would like to know for each pair of positions (A, B) which of the 4 combinations of amino acids present at A and B maximize the activity. For 19 substitutions there are a total of 171 total possible pairs to consider (: A with B, A with C, A with D,...B with C, B with D, etc). Each pair consists of 4 possible states: both wild-type, both substituted and 2 possibilities in which only 1 is substituted. Thus to perform one separate test for each possible combination in every pair would require 684 variants. To test each of these combinations in at least 3 different sequence contexts could require >2,000 variants, which would be quite impractical. Since computational resources are cheap we instead used the 1000 subsamples of the training sets to "virtually" test which pairs of substitutions led to better predictions by our algorithms.

To do this we expanded one amino acid pair at a time into its four possible combinations and optimized the new weight vector. Thus, for each of the 171 possible position pairs (A, B), we built a model from each of the 1000 subsamples using one weight for each position except for the (A, B) pair for which we used 4 weights: one for each possible combination of the substitutions at position A and B. We computed the loss of the linear models on predicting activities of the 5 variants held out from each subsample (the loss quantifies the differences between the predicted and measured activities) and averaged the loss over the 1000 subsamples. If one or more pairs of substitutions improved the model (ie reduced the mean loss) we fixed the pair that produced models with lowest mean loss and then repeated the process. Each time we picked the pair of positions that produced the largest reduction in the average loss. We stopped expanding amino acid pairs when no further reduction of the mean loss occurred.

Examples of weights calculated by considering amino acid pairs are shown in Figure 3B. A comparison of Figure 3A with 3B shows that the expansion of 3 amino acid pairs into 4 combinations produced a model that also modified the weights of the single substitutions. Thus S123A goes from being less than 1 standard deviation above zero to more than 1 standard deviation above zero. One substitution (Y194S) goes from being very positive to negative (green circles in Figure 3). This substitution was only represented twice in active variants in the training set, and both of these variants had very low activities (variants 1–12 and 1–35; Additional file 2). The consequences of these weights for variant design are discussed in sections 3.2 and 3.3.

#### 2.8 Design and testing of variant set 3

In designing variant set 3 we aimed to obtain further increases in proteinase K activity. We also wanted to test whether new variant designs were improved either by accounting for the general context dependence of a substitution, or by considering epistatic interactions. Variant set 3 was therefore designed in 3 parts to answer these 3 questions.

Our first two designs used only linear models. We selected one sequence for each algorithm by combining substitutions whose weights were calculated by that algorithm to be greater than zero (variants 3-1 to 3–5, design method J in Additional file 2). We selected a second sequence for each algorithm by combining substitutions whose weights were calculated by that algorithm to be at least 1 standard deviation greater than zero (variants 3–6 to 3–9, design method K in Additional file 2). Values for the mean and standard deviations for substitution weights calculated by each method are shown in Table 2.

The third design used models that considered amino acid pairs. We selected one sequence for each algorithm by combining substitutions or substitution pairs whose weights were calculated by that algorithm to be at least 1 standard deviation greater than zero (variants 3–10 to 3–16, design method L in Additional file 2). When more than one pair had a positive substitution weight, we selected the pair with the highest value when the standard deviation was subtracted from the mean. Thus in Figure 3B we chose 337S, 355P over S337N, 355P and S337N, P355S although the weights for all three combinations were more than 1 standard deviation above zero. As for designs from the linear models, we only included a combination for a pair if that combination was present in the training set at least 3 times. Thus in Figure 3B we rejected I132V, 208K because it was present only twice, but instead chose 132IK, 208H which had a slightly higher value than 132I, 208K.

**Figure 4:**
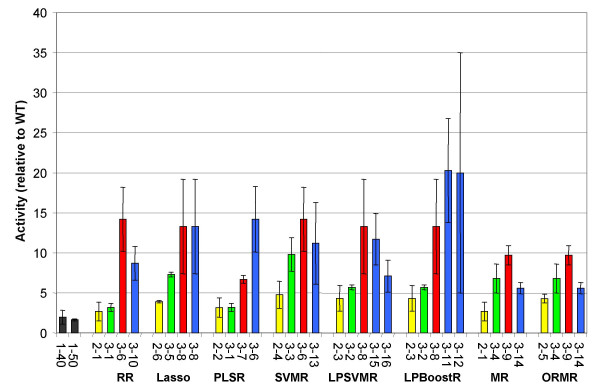
**Activities of variants designed using substitution weights**. Activities towards N-Succinyl-Ala-Ala-Pro-Leu p-nitroanilide were measured at 37°C following a 5 minute heat treatment of the enzyme at 68°C. Activities are expressed relative to the mean activity of duplicates of wild-type proteinase K. Error bars represent one standard deviation of the activity measurements. Variants are grouped according to the machine learning algorithm used to calculate substitution weights (indicated below each group), and are compared with the best variants from the initial design set (variants 1–40 and 1–50 black bars, on the left). The first design (yellow bars, design method G in Additional file 2) of each group belongs to set 2. We included a substitution in the design if it occurred at least three times in the training set and its mean weight was at least one standard deviation above zero. All remaining designs in each group belong to set 3. The second in each group (green bars, design method J in Additional file 2) includes substitutions occurring at least three times and whose mean weights were merely positive (eg Figure 3A, red and blue circles). The third in each group (red bars, design method K in Additional file 2) contained all substitutions occurring at least three times and whose mean weight was at least one standard deviation above zero (eg Figure 3A, red circles). Note that this third design in each group is always better than the second. The last variant(s) in each group (blue bars, design method L in Additional file 2) were designed by modeling interdependent substitutions (eg Figure 3B, red circles).

For every machine learning algorithm, the design that incorporated substitutions only when the mean substitution weights were at least 1 standard deviation above zero, outperformed the design that incorporated substitutions when the mean substitution weights were simply greater than zero. There was no clear pattern when epistatic models were used: the data is shown in Figure 4 and discussed in more depth in section 3.3.

### 3. Analysis of the design methods

#### 3.1 Functional contributions of amino acid substitutions

Ten of the initial set of 24 substitutions that we selected had a beneficial effect on proteinase K activity, a success rate of 40%. The substitutions were selected from a total of more than 7,000 possible (371 positions × 19 alternatives at each position), by using alignments of homologous sequences without the use of any structural information.

Table 1 shows the effect of each of substitution next to the method used to select it. Two of the 24 substitutions selected (Y151A and G293A) were selected as positive by all 8 algorithms in the second analyses (section 2.7). These substitutions were incorporated into every variant in round 3 and are present in the most active variants. Both of these substitutions were chosen from alignments of thermostable homologs of proteinase K. In all, eight of the ten positive substitutions (S123A, I132V, Y151A, L180I, S273T, G293A, K332R and S337N) were selected based on the presence of the new amino acid in an alignment of thermostable homologs. By contrast, four of the five substitutions that were not found in any active variant were designed based on literature reports of their stabilizing effects in subtilisin (N95C, P97S, E138A, and L299C)[34,35]. We were thus most successful in choosing beneficial substitutions by selecting changes that occur in homologous natural proteins.

The positions of all substitutions used are shown mapped onto the structure of proteinase K [see Additional files 3, 4 and 5]. We could see no obvious pattern distinguishing the locations of beneficial from detrimental substitutions, nor were we able to identify simple structural reasons for the effect of the substitutions.

For future extensions of this method, if a target activity is not achieved with the initial set of substitutions, additional substitutions can be chosen and incorporated into a new set of variants along with the best substitutions that have already been tested. Results from previous experiments can improve a second cycle of substitution selection. For example, to obtain further improvements in proteinase K activity by incorporating new substitutions, we would pick more substitutions that appear in alignments of thermostable homologs and avoid those reported to confer stability on subtilisin. More data from other systems will be required to determine whether the best method for picking substitutions varies depending on the protein target or the desired application. In either case, using a variety of methods for the initial selection, then analyzing the functional contributions of substitutions selected by different methods is likely to provide a good starting point for other protein engineering projects.

#### 3.2 Representation of substitutions in the training data set

In our initial set of variant designs (section 2.3) we aimed to have each amino acid substitution represented more than 6 times. Because so many of our random combinations of substitutions were inactive this resulted in a training set where different substitutions were represented very unevenly.

There are two major consequences of underrepresentation of a substitution for machine learning analysis. One can be seen in Figure 3A, where the MR algorithm assigned Y194S a high weight even though both variants in the training set have very low activity. Because there are only 2 active variants encoding Y194S, the machine learning algorithms tend to assign weights to the substitution that improve the fit of other substitutions to the model, but do not really reflect the contribution of the underrepresented substitution. The more the substitution is represented the less likely this is to occur because there are more datapoints that the weight has to be consistent with. Table 2 also shows that this phenomenon is dependent on the machine learning algorithm used. Two of the three algorithms that use one-norm regularization (Lasso and LPSVMR) and use fewer larger weights to fit the data give very low scores to Y194S.

A second consideration that arises when a substitution is underrepresented is that it is difficult to assess effects of context upon the contribution of a substitution. Sometimes a substitution may be beneficial with one set of other substitutions, but deleterious with a different set. The fewer times a substitution has been tested, the less likely such interactions are to be detected.

In this study we required that a substitution occur at least 3 times for us to use it in a subsequent design. For future designs of variant sets it will be important to ensure that each substitution is adequately represented in the training data set.

#### 3.3 Accounting for interactions between amino acid substitutions

The extent to which one substitution affects the contribution of another substitution to protein function is difficult to predict. For proteins that have evolved by the sequential accumulation of point mutations, most of those mutations must work well in many different contexts. This is because each new mutation will produce a new sequence context, so an enzyme whose amino acids were predominantly very context dependent would be largely immutable. This view is supported by a study in which all amino acid differences in 15 natural subtilisins were recombined by DNA shuffling [49]. Almost all possible pairwise combinations of amino acid differences were found in functional subtilisin enzymes produced by this recombination, suggesting that amino acid covariation seen in the original 15 orthologs resulted from common ancestral derivation rather than functional constraints. Selecting amino acid substitutions that occur in natural homologs should therefore provide a useful bias towards variations that are tolerated in many contexts.

Different subsamples of the training set produced different values for the weights of each substitution. This difference probably arises from noise in the data as well as from possible context effects. To accommodate this variation in our designs we used the standard deviation of each weight as a measure of its variability of effect. We compared the activities of third cycle variants designed by combining all substitutions whose mean weights were positive (substitution weight example shown in Figure 3A blue and red circles, activities shown in Figure 4 variants 3-1 to 3–5, green bars), with those designed by combining only substitutions whose mean weight was more than 1 standard deviation above zero (substitution weight example shown in Figure 3A red circles only, activities shown in Figure 4 variants 3–6 to 3–9, red bars). For every machine learning algorithm, the variant that contained only substitutions whose mean weights were at least 1 standard deviation above zero was more active than the corresponding variant that included all substitutions with positive weights. The standard deviation of a substitution weight thus appears to provide a useful evaluation of the likely contribution of that substitution to protein function.

As described in sections 2.7 and 2.8 we also tested designs based on regression models that considered epistatic amino acid interactions. Activities of variants designed in this way are also shown in Figure 4 (variants 3–10 through 3–16, blue bars, substitution weight example in Figure 3B). Only the algorithms PLSR and LPBoostR produced more active designs based on modeling amino acid interactions than the corresponding designs produced when all substitutions were modeled independently. One of these, LPBoostR, found the most active of all the 95 sequences we tested (variants 3–11 and 3–12). However we note that different machine learning algorithms selected different amino acid pairs for expansion. It is therefore unclear to us whether these pairings are actually related to epistatic interactions between the amino acids themselves, or result from differences in the machine learning methods' ways of minimizing discrepancies between measured and predicted activities in a small and unevenly distributed dataset.

Understanding interactions between specific pairs of amino acid substitutions is unlikely to limit the protein engineering method described here. To test every combination of all pairs of amino acid substitutions would rapidly become prohibitively expensive: for 19 substitutions it would require more than 2000 variants (see section 2.7). However it is relatively simple to instead select substitutions that work well in many contexts and to reject those that work well in some contexts but poorly in others. This can be done by using the standard deviation of the substitution weight over many subsamples of the training set, keeping only those whose mean weights are more than one standard deviation above zero. This will also ensure that if additional substitutions are incorporated subsequently, those substitutions already accepted and fixed are likely to be generally tolerant to further change.

#### 3.4 Comparison with other design methods

**Figure 5:**
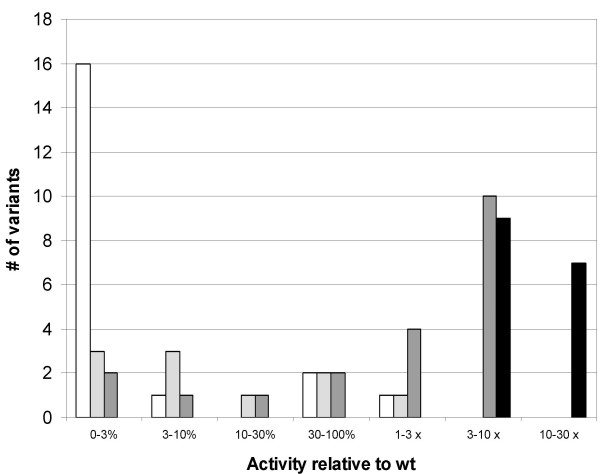
**Machine learning design compared with random choices and "expert" designs**. Distribution of activities of 4 sets of variants designed using different methods are shown. Set A (white bars, variants 1–2, 1–6, 1–12, 1–13 and 1–34 to 1–49, total of 20 variants) contain arbitrarily selected combinations of 3, 5 or 6 substitutions. Set B (light shading, variants 1–50 to 1–59, total of 10 variants) were designed by manual analysis of the sequence and activity data from variants 1 through 49. Set C (dark shading, variants 2-1 to 2–20, total of 20 variants) were designed using machine learning algorithms based on the data from variants 1 through 59. Set D (black fill, variants 3-1 to 3–16, total of 16 variants) were designed using machine learning algorithms based on the data from variants 1-1 through 1–59 and 2-1 through 2–20.

As a control to determine whether the same degree of activity improvement could be achieved by simpler means, we analyzed the activity distribution for 4 sets of variants. The first set, taken from the first 49 variants synthesized, comprised 20 variants which contained arbitrarily selected combinations of the 19 substitutions considered in the machine learning designs (1–2, 1–6, 1–12, 1–13 and 1–26 through 1–49; Figure 5, white bars). The second set comprised 10 variants that were designed by our "expert" analysis of the sequence and activity data from the first 49 variants (1–50 through 1–59; Figure 5, light shading). The third and fourth sets comprised 20 and 16 variants designed using machine learning analysis (2-1 through 3–16; Figure 5, dark shading and black fill respectively). The activities of the randomly designed variants are predominantly extremely low: 80% are less than 3% of wild type activity and just one is more active than wild type. The activities for the variants designed by manual data analysis are a little more evenly distributed, but still only one is more active than wild type. By comparison 70% of the variants designed in the first cycle of machine learning were more active than wild type and all of the variants designed in the second cycle of machine learning were at least 3-fold more active than wild type. While it is not possible for us to compare machine learning with all available protein engineering methods, this control shows that machine learning identified highly functional combinations of substitutions that could not be readily obtained either by random selection or by manual analysis.

The machine learning designs, which resulted in enzyme activity increases of up to 20-fold, differed from classical experimental designs (see for example [9]) because of the epistatic effect of some amino acid changes. Amino acid changes or pairs of changes that completely eliminate protein activity will mask any positive or negative contributions made by other substitutions that occur with them. Experimental designs such as Taguchi matrices [50,51] minimize the number of experiments by combining many variables at once. A Taguchi orthogonal design for testing 24 substitutions would have produced 48 variants containing different combinations of 12 substitutions and 1 containing all 24. Our initial design incorporated only 6 changes into each of 24 variants. Although this was a less complete testing of the combinations, because 5 substitutions abolished enzyme activity, we did obtain 4 variants with detectable activity. By synthesizing an additional 8 variants we were able to identify the 19 functional amino acids in our set of substitutions. By contrast the Taguchi design would almost certainly have produced only inactive variants and thus no information.

Machine learning has also been used in the related domain of drug design to search large libraries of small molecules for compounds with maximal activity towards a biological target. The activity levels of some compounds are known and "active learning" methods [52] are used to select the next batch of compounds to be tested [53]. There are a number of significant differences between these searches and those in a protein engineering setting. For example small molecules are described by feature vectors of sizes between 10 and 10^5 ^[54], while each protein variant in this study is described by 24 binary features. Another difference is that in drug discovery large datasets are available for testing various machine learning methods [53,55] while no such data exists for proteins. Small molecule datasets are also generally quite large, typically with 10^3 ^to 10^4 ^compounds: Fang *et al *estimate that training sets of 10,000 member compounds are required to build a predictive model but in this study we tested a total of less than 100 variant proteins. In part this is possible because our protein descriptors are so much simpler and the relatedness of any pair of variants is unambiguous. This allowed us to identify improved proteins and then to focus on highly related proteins.

#### 3.5 Multiple protein properties are modified simultaneously

The activity of proteinase K that we targeted depends on activity towards the substrate and heat-stability of the protein. We were interested in knowing whether we had modified one or both of these properties. A second motivation was that we were unable to measure the concentration or proteinase K: it autodigested so efficiently that we were unable even to visualize it on a gel. Since the half-life of the protein at 68°C should be essentially independent of the protein concentration, changes in half-life reflect changes in the protein itself and not possible influences of expression levels.

**Figure 6:**
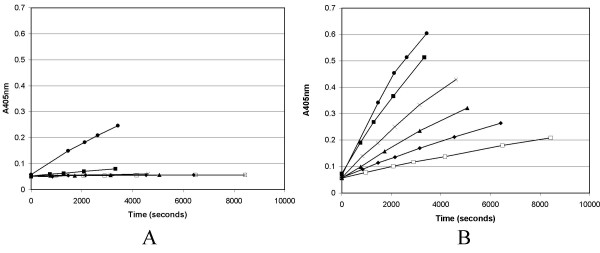
**Increases in proteinase K activity with and without heating**. Proteinase K variants were tested from triplicate independent cultures for activity after heating at 68°C for different times: unheated (circles), 2.5 minutes (squares), 5 minutes (crosses), 7.5 minutes (triangles), 10 minutes (diamonds) and 15 minutes (open squares). A: absorbance at 405 nm of substrate incubated with wild type proteinase K, B: absorbance at 405 nm of substrate incubated with variant 3–9.

**Figure 7:**
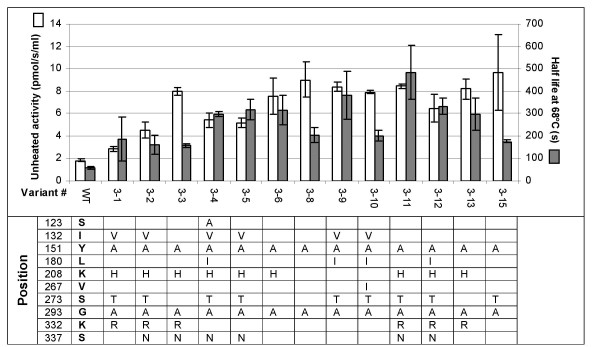
**Changes in activity and half-life in designed protein variants**. Activity (unheated) and half life were calculated for 13 protein variants and wild type proteinase K. The activity without heating was calculated from the initial slopes of the A_405 _measurements without heating (white bars), examples shown in Figure 6. The half-life at 68°C (shaded bars) was calculated using the initial slopes after different heating times and fitting to an exponential curve. Error bars represent one standard deviation of the experimental measurements. The wild-type values are shown on the left. The substitutions of each variant are given in the column below the variant name. Only 10 of the 19 positions are shown. In the remaining 9 positions, all variants contained amino acids from the wild-type sequence.

We measured the activity towards the substrate and the half-life at 68°C of 13 of the best variants. Figure 6A shows the activity of wild type proteinase K following different exposures to 68°C, Figure 6B shows one of the third cycle variants (3–9) after the same heating times. Figure 7 shows the activity without heating (white bars) and the half-life (shaded bars) for wild type proteinase K and 13 third cycle variants, as well as the substitutions in each variant. With combinations drawn only from a small set of 24 selected substitutions, a significant diversity of functional combinations provided the desired outcome, from variants in which the primary effect was increasing overall activity (3-3) to those in which both activity and half-life were improved (3–11). We expect that this pattern would continue if we attempted to deconvolute further. For example, the increase in activity without heat treatment is probably a combination of increased specific activity and increased protein expression levels, with varying contributions from each activity in each variant. Most importantly for the approach described here, several properties were altered simultaneously to improve an activity that depended on multiple properties.

#### 3.6 Different machine learning predictions from different data sets

Different parts of variant set 2 were designed using different data sets (see section 2.6). Variants 2-1 to 2–12 were designed using the activity of the first set of protein variants after heat-treatment, while variants 2–13 to 2–20 used the activity without heat treatment. Variants 2–13 to 2–20 contained new combinations of the substitutions incorporated into 2-1 to 2–12, as well as 2 that were not included using only the heated data (S273T and V167I; Additional file 2). Five variants designed using the unheated data were more active than wild type, approximately the same proportion as those that were designed using the activities after heating (Additional file 2).

Although some variants designed using the unheated activities were active after heating, the activities without heating were in no way intended as a surrogate for the activity after heating. We used the unheated activities only to obtain a larger set of sequences, but did not measure the activities of variant sets 2 or 3 without heating. There are clearly combinations of substitutions that produce increased activity over wild type without heat treatment, but lower activity than wild type after heating (eg 1–13, 1–30, 1–37, 1–43 and 1–47). If we had performed several cycles of engineering using only the unheated activities, we would therefore expect only a subset to be active after heating.

#### 3.7 Differences in predictions of the machine learning algorithms

The different machine learning algorithms did not converge to the same sequence design. The eight algorithms produced 4 different variant designs (3–6 through 3–9) using the same training data set. These differences in turn arose from differences in calculated mean and standard deviations for the substitution weights (shown in Table 2), which themselves resulted from differences in the way in which the algorithms model the data. The activities of the variants designed using different machine learning algorithms were very comparable (see Figure 4), and we were unable to really distinguish between them by their performances. The comparable performance of all the machine learning algorithms we used is probably due to the fact that we have too few example proteins. We expect that with more examples, clear differences between the algorithms could appear. Testing this hypothesis will require analysis of additional datasets.

It is unclear from the activity data whether there is a single optimal sequence, although there are clearly many improved sequences. For example the two most thermostable variants, 3–9 and 3–11, share 3 substitutions but differ at 5 positions (see Figure 7). The substitutions I132V and L180I appear in 3–9 but not in 3–11. Addition of either of these 2 substitutions to 3–11 leads to variants with lower thermostability than either 3–9 or 3–11 (variants 3–2 and 3–12). Thus the effect of these two substitutions appears to be influenced by other changes in the protein. This context dependence of substitutions suggests a limitation for approaches such as site saturation mutagenesis [56], in which all changes are considered independently and then combined based only on their behaviour in the wild-type context.

## Conclusion

We have developed a new synthetic biology approach to protein engineering in which amino acid substitutions are selected, incorporated in different but defined combinations into a small number of variant enzymes which are individually synthesized and tested functionally. Machine learning algorithms are then used to assign values to the functional contribution of each substitution, which serves as the basis for a further set of variant designs. The process is repeated until a target activity is achieved. We have tested the approach using proteinase K as a target protein.

Substitutions that improved the activity of proteinase K were primarily identified using alignments of naturally occurring homologous proteins, structural information was not used. The exponential accumulation of natural DNA sequences [57,58] could facilitate the use of phylogenetic information for substitution selection in many other systems, helping to remove the prerequisite of obtaining high resolution crystal structures before initiating a protein engineering project.

We tested 8 different machine learning algorithms and found them all able to produce predictive models describing the contributions of individual amino acid substitutions to the activity of proteinase K. We also found that it was unnecessary to consider all possible amino acid interactions to obtain substantial improvements in protein activity. However, it was advantageous to use the machine learning models to identify (and eliminate) substitutions whose effect appeared to vary significantly depending on the sequence context.

By designing, synthesizing and testing a total of only 95 specific proteinase K variants, of which 36 were designed using machine learning algorithms, we obtained a 20-fold increase in protein activity. Application of the strategy described here to other systems should allow proteins to be optimized using functional measurements for small numbers of protein variants. This would obviate the need for library construction and high throughput screening. Instead, variants could be directly tested in complex low-throughput assays that accurately reflect the combination of properties desired for the final application of the optimized protein.

## Methods

### Gene synthesis

A proteinase K-encoding gene was designed using Protein-2-DNA software [30] to select a codon distribution mimicking natural highly expressed *E. coli *proteins [31]. The gene was assembled from chemically synthesized oligodeoxyribonucleotides (purchased from Operon) as described previously [59,60], and cloned into pBAD/gIII (Invitrogen). Variant genes were synthesized by replacing oligonucleotides encoding the amino acids with those encoding the desired amino acid substitutions. Variants were cloned between the *NcoI *and *SalI *sites of the sequence [see Additional file 1].

### Proteinase K expression and purification

Proteinase K was expressed in the *E. coli *periplasm and purified on Ni-NTA. Briefly, a single colony of *E. coli *carrying a proteinase K variant in pBAD/gIII was picked from a carbenicillin plate and grown overnight. Forty microliters of culture was then diluted into 4 ml pre-warmed LB-carbenicillin, grown for 3 to 4 hours (to an A_600 _of 0.2–0.3), arabinose was added to 0.2% w/v and the cells were grown overnight. All growth was performed at 30°C in LB containing 50 μg/ml carbenicillin. Cells were pelleted in a microfuge, thoroughly resuspended in 200 μl of 20% wv sucrose, 200 mM NaH_2_PO_4_, pH 7.4, 1 mM EDTA and 30 U/μl lysozyme (freshly added from a 30,000 U/μl stock: Ready-Lyse, Epicentre) and incubated at 25°C for 5 minutes. Cells were subjected to osmotic shock by addition of 200 μl ice-cold water, mixed by inversion and incubated for 5 minutes on ice to release periplasmic protein. Cells were pelleted in a microcentrifuge. The supernatant was removed, adjusted to 300 mM NaCl, 10 mM imidazole, 67 mM NaH_2_PO_4_, pH 7.4 and loaded onto an Ni-NTA column (Qiagen) pre-equilibrated with 50 mM NaH_2_PO_4_, pH 7.4, 300 mM NaCl, 10 mM imidazole. The column was washed twice with 600 μl of 50 mM NaH_2_PO_4_, pH 7.4, 300 mM NaCl, 20 mM imidazole and then eluted twice with 100 μl of 50 mM Tris-Cl pH 7.4, 300 mM NaCl, 250 mM imidazole. The two eluates for each culture were pooled.

### Proteinase K activity measurements

Proteinase K Ni-NTA eluates were heat-treated in a PCR machine at 68°C for 5 minutes. Proteinase K activity was measured by addition of 10 μl Ni-NTA eluate to 90 μl reaction buffer. Final reaction conditions were 50 mM Tris-Cl pH 7.4, 180 mM NaCl, 5 mM CaCl_2_, 25 mM imidazole, 500 μM N-Succinyl-Ala-Ala-Pro-Leu p-nitroanilide substrate (Sigma S-8511). The reaction was incubated at 37°C, and followed by measuring absorbance at 405 nm. Activities were calculated from the initial rate of reaction and comparison with a standard curve constructed using 4-nitroanilide [61-63]. Because proteinase K self-digests we were unable to accurately determine protein concentration. Activities are therefore expressed either relative to the wild-type enzyme activity, or as pmol substrate hydrolyzed per second per ml of initial culture from which the proteinase K variant was purified (pmol/s/ml). The activities measured for each sequence in Set 1 and Set 2 are shown in Additional file 2.

### Proteinase K half-life calculations

Proteinase K Ni-NTA eluates were heat-treated in a PCR machine at 68°C for 0, 2.5, 5, 7.5, 10 and 15 minutes. Proteinase K activity was measured at 37°C, and followed by measuring absorbance at 405 nm. Initial reaction rates were plotted against heating time and fitted to an exponential curve.

### Machine learning algorithms

Machine learning algorithms and methods used for variant design are detailed in Additional file 1. Variant set 1 (active variants in Additional file 2) was the training data set used for the design of set 2. Multiple measurements of the same variant were treated as separate pairs. All algorithms were implemented using commercially available software (Matlab from Mathworks).

## Authors' contributions

JL performed the machine learning analyses and new variant designs. MKW guided the machine learning analysis and new variant designs and helped to draft the manuscript. SG selected the initial amino acid substitutions, designed the first set of variants, designed synthesis strategies for all variants and participated in development of new variant designs. JEN synthesized genes and developed the protein purification and assay. RPW synthesized genes and analyzed their sequences. CG participated in experimental designs and in development of new variant designs and helped to draft the manuscript. JM synthesized genes, performed activity measurements, participated in experimental designs and in development of new variant designs and drafted the manuscript. All authors read and approved the final manuscript.

## Supplementary Material

Additional File 1 Supporting Material. Contains DNA and amino acid sequence of proteinase K, legends for structure figures (additional files 3, 4 and 5) and details of machine learning methods. Click here for file

Additional File 2 Table 2. Contains sequence and activity information for all variants tested in this study. Click here for file

Additional File 3 Supporting Material Figure 2. Positions of amino acid substitutions mapped onto the structure of proteinase K. The image should be opened using Swiss Protein Data Bank Viewer [64]. Click here for file

Additional File 4 Supporting Material Figure 3. Positions of amino acid substitutions mapped onto the structure of proteinase K. Click here for file

Additional File 5 Supporting Material Figure 4. Positions of amino acid substitutions mapped onto the structure of proteinase K. Click here for file

## Figures and Tables

**Table 1:** Amino acid substitutions selected for modification of proteinase K.

**Substitution**	**Effect**	**Reason for Selection**
N95C	Lethal	Literature report: disulphide bond between 95C and 299C reported to stabilize subtilisin BPN' (S3C and Q206C in subtilisin)[34,35].
P97S	Lethal	Literature report: P to S reported to increase stability in subtilisin BPN' (P5S in subtilisin)[34,35].
S107D	Negative	Homolog sequence alignment analysis: D present at this position in 2/42 Group B homologs.
S123A	Positive	Thermostable homolog sequence alignment analysis: residue found in 8/11 Group C homologs and 6/42 Group B homologs.
I132V	Positive	Thermostable homolog sequence alignment analysis: residue found in 10/11 Group C homologs, 1/6 Group A homologs and 13/42 Group B homologs. Also a favorable change according to Dayhoff substitution matrix[36].
E138A	Lethal	Literature report: acidic residue to A reported to increase stabibility in subtilisin BPN' (D41A in subtilisin)[34,35].
M145F	Negative	Literature report: M to F reported to increase stabibility in subtilisin BPN' (M50F in subtilisin) [34,35].
Y151A	Strong positive	Thermostable homolog sequence alignment analysis: residue found in close thermostable homolog gi|131084 and 2/42 Group B homologs.
V167I	Negative	Substitution matrix-derived change: favorable change according to Dayhoff substitution matrix [36]. Residue found in 1/6 Group A homologs and 27/42 Group B homologs.
L180I	Positive	Thermostable homolog sequence alignment analysis: residue found in 10/11 Group C homologs, 1/6 Group A homologs and 10/42 Group B homologs. Also a favorable change according to Dayhoff substitution matrix [36].
Y194S	Negative	Random mutation obtained during synthesis of wt proteinase K.
A199S	Negative	Substitution matrix-derived change: favorable change according to Dayhoff substitution matrix [36]. Residue found in 1/6 Group A homologs and 9/42 Group B homologs.
K208H	Positive	PCA identification of amino acids responsible for clustering of thermophilic sequences gi|4092486; gi|56160990; gi|114081 within Group A and B homologs [37].
A236V	Lethal	PCA identification of amino acids responsible for clustering of thermophilic sequences gi|4092486; gi|56160990; gi|114081 within Group A and B homologs [37].
R237N	Negative	Thermostable homolog sequence alignment analysis: residue found in 9/11 Group C homologs, 1/6 Group A homologs and 1/42 Group B homologs.
P265S	Negative	Structural considerations: literature report: P5S reported to increase stability in subtilisin BPN' (P5S in subtilisin) [34,35]. 265S found at this position in proteinase K closest homolog (gi|131084).
V267I	Positive	Substitution matrix-derived change: favorable change according to Dayhoff substitution matrix [36]. Residue found in 1/6 Group A homologs and 1/41 Group B homologs.
S273T	Positive	Thermostable homolog sequence alignment analysis: residue found in 11/11 Group C homologs, 1/6 Group A homologs and 29/41 Group B homologs. Also a favorable change according to Dayhoff substitution matrix [36].
G293A	Strong positive	Thermostable homolog sequence alignment analysis: residue found in 11/11 Group C homologs, 1/6 Group A homologs and 38/41 Group B homologs.
L299C	Lethal	Disulphide bond between 95C and 299C reported to stabilize serine proteases [34,35].
I310K	Negative	Literature report: K substitution at this position reported to increase stabibility by adding hydrogen bonding in subtilisin BPN' (Y217K in subtilisin) [34,35].
K332R	Positive	Thermostable homolog sequence alignment analysis: residue found in 8/11 Group C homologs and 1/6 Group A homologs. Also a favorable change according to Dayhoff substitution matrix [36].
S337N	Positive	Thermostable homolog sequence alignment analysis: residue found in 8/11 Group C homologs, 1/6 Group A homologs and 2/41 Group B homologs. Also a favorable change according to Dayhoff substitution matrix [36].
P355S	Negative	Structural considerations: literature report: P5S reported to increase stability in subtilisin BPN' (P5S in subtilisin) [34,35]. 355S found at this position in proteinase K closest homolog (gi|131084).

Selection criteria and references are shown for 24 amino acid substitutions within proteinase K. Group A, wild type plus 5 closest homologs (>90% identity); Group B, 42 homologs (30–90% identity); Group C, 11 thermostable homologs. The effect of each substitution is also shown. Lethal: no active variant contained this substitution. Negative: the substitution was not selected by any of the third round design methods. Positive: the substitution was selected by at least one third round design method and was present in at least one third round variant with activity > 3× wild type. Strong positive: the substitution was selected by all third round design methods and are present in the most active variants.

**Table 2:** Vector weights calculated for amino acid substitutions.

	**RR**		**Lasso**		**PLSR**		**SVMR**	
**Substitution**	**M**	**σ**	**M**	**σ**	**M**	**σ**	**M**	**σ**

S107D	-0.03	0.13	0.00	0.02	-0.70	0.26	-0.16	0.13
S123A	-1.00	0.13	-0.41	0.35	-1.42	0.23	-0.93	0.14
I132V	0.04	0.44	0.04	0.55	0.32	0.76	-0.34	0.29
M145F	-1.46	0.19	-2.27	0.49	-1.98	0.32	-1.58	0.20
Y151A	1.18	0.23	0.91	0.23	1.66	0.37	0.91	0.15
V167I	-0.97	0.13	-1.09	0.15	-1.10	0.17	-0.79	0.14
L180I	-0.23	0.15	-0.05	0.10	-0.35	0.19	-0.36	0.13
Y194S	0.27	0.20	0.00	0.01	0.94	0.73	0.01	0.14
A199S	-1.16	0.39	-1.09	0.46	-2.66	0.98	-0.86	0.21
K208H	0.28	0.15	0.07	0.12	0.52	0.18	0.36	0.17
R237N	-0.93	0.09	-0.91	0.13	-1.21	0.12	-0.86	0.15
V267I	-0.48	0.11	-0.32	0.14	-0.68	0.13	-0.16	0.12
S273T	0.12	0.14	0.01	0.06	0.28	0.19	-0.05	0.17
G293A	1.95	0.13	2.24	0.14	2.10	0.17	1.70	0.13
K332R	0.07	0.13	-0.01	0.05	0.02	0.14	0.09	0.15
S337N	-0.02	0.14	0.03	0.09	-0.20	0.15	0.03	0.14
P355S	-1.08	0.12	-1.20	0.15	-1.25	0.13	-1.10	0.15

	**LPSVMR**		**LPBoostR**		**MR**		**ORMR**	

**Substitution**	**M**	**σ**	**M**	**σ**	**M**	**σ**	**M**	**σ**

S107D	-0.01	0.20	-0.02	0.21	-0.35	0.24	-0.35	0.24
S123A	-0.41	0.43	-0.40	0.41	0.52	0.93	0.52	0.93
I132V	0.22	0.69	0.18	0.56	2.61	0.91	2.61	0.91
M145F	-2.39	0.53	-2.39	0.53	-5.33	1.05	-5.33	1.05
Y151A	0.82	0.24	0.82	0.25	0.64	0.24	0.64	0.24
V167I	-0.99	0.24	-0.98	0.23	-1.63	0.24	-1.63	0.24
L180I	-0.20	0.16	-0.19	0.16	0.60	0.23	0.60	0.23
Y194S	-0.02	0.08	0.21	0.38	4.59	2.10	4.59	2.10
A199S	-0.49	0.33	-0.73	0.54	-4.92	2.03	-4.92	2.03
K208H	0.16	0.18	0.14	0.18	0.01	0.13	0.01	0.13
R237N	-0.96	0.29	-0.96	0.29	-1.59	0.57	-1.59	0.57
V267I	-0.41	0.23	-0.41	0.23	-1.33	0.14	-1.33	0.14
S273T	0.19	0.33	0.15	0.28	0.96	0.58	0.96	0.58
G293A	2.18	0.25	2.20	0.25	3.20	0.14	3.20	0.14
K332R	0.12	0.19	0.14	0.21	-0.33	0.13	-0.33	0.13
S337N	0.27	0.28	0.26	0.28	0.34	0.59	0.34	0.59
P355S	-1.34	0.35	-1.35	0.34	-1.95	0.57	-1.95	0.57

Mean (M) and standard deviation (σ) values are shown for the 19 substitutions for which weights were calculated using machine learning. The values were calculated from 1000 subsamples of the variants with measurable activity from sets 1 and 2, where 5 variant sequences were randomly omitted from each subsample.
