# Supplementary material for: Engineering proteinase K using machine learning and synthetic genes
Source: BMC Biotechnol. 2007 Mar 26;7:16. doi: 10.1186/1472-6750-7-16 (PMC1847811; doi:10.1186/1472-6750-7-16)
Supplement: Additional File 1 — Supporting Material. Contains DNA and amino acid sequence of proteinase K, legends for structure figures (additional files 3, 4 and 5) and details of machine learning methods. [file 1472-6750-7-16-S1.doc]

# Engineering proteinase K using machine learning and synthetic genes

**SUPPORTING MATERIAL**

**Proteinase K optimized for expression in *E. coli***

The sequence of synthetic "wild-type" proteinase K is shown in Supporting Material Figure 1. The 15 amino acid leader sequence from the original *Tritirachium album* protein [1] was replaced by an 18 amino acid bacteriophage f1 gene III leader peptide [2]. A hexahistidine tag was added to the C-terminus. The entire protein was back-translated with an *E. coli* codon bias [3, 4].

taacaggaggaattaaccatgaaaaaactgctgttcgcgattccgctggtggtgccgttc

M K K L L F A I P L V V P F

tatagccatagcaccatggcaccggccgttgaacagcgttctgaagcagctcctctgatt

Y S H S T M A P A V E Q R S E A A P L I 14

gaggcacgtggtgaaatggtagcaaacaagtacatcgtgaagttcaaggagggttctgct

E A R G E M V A N K Y I V K F K E G S A 34

ctgtctgctctggatgctgctatggaaaagatctctggcaagcctgatcacgtctataag

L S A L D A A M E K I S G K P D H V Y K 54

aacgtgttcagcggtttcgcagcaactctggacgagaacatggtccgtgtactgcgtgct

N V F S G F A A T L D E N M V R V L R A 74

catccagacgttgaatacatcgaacaggacgctgtggttactatcaacgcggcacagact

H P D V E Y I E Q D A V V T I N A A Q T 94

aacgcaccttggggtctggcacgtatttcttctacttccccgggtacgtctacttactac

**N** A **P** W G L A R I S S T **S** P G T S T Y Y 114

tacgacgagtctgccggtcaaggttcttgcgtttacgtgatcgatacgggcatcgaggct

Y D E S A G Q G **S** C V Y V I D T G **I** E A 134

tctcatcctgagtttgaaggccgtgcacaaatggtgaagacctactactactcttcccgt

S H P **E** F E G R A Q **M** V K T Y Y **Y** S S R 154

gacggtaatggtcacggtactcattgcgcaggtactgttggtagccgtacctacggtgtt

D G N G H G T H C A G T **V** G S R T Y G V 174

gctaagaaaacgcaactgttcggcgttaaagtgctggacgacaacggttctggtcagtac

A K K T Q **L** F G V K V L D D N G S G Q **Y** 194

tccaccattatcgcgggtatggatttcgtagcgagcgataaaaacaaccgcaactgcccg

S T I I **A** G M D F V A S D **K** N N R N C P 214

aaaggtgttgtggcttctctgtctctgggtggtggttactcctcttctgttaacagcgca

K G V V A S L S L G G G Y S S S V N S A 234

gctgcacgtctgcaatcttccggtgtcatggtcgcagtagcagctggtaacaataacgct

A **A** **R** L Q S S G V M V A V A A G N N N A 254

gatgcacgcaactactctcctgctagcgagccttctgtttgcaccgtgggtgcatctgat

D A R N Y S P A S E **P** S **V** C T V G A **S** D 274

cgttatgatcgtcgtagctccttcagcaactatggttccgtcctggatatcttcggccct

R Y D R R S S F S N Y G S V L D I F **G** P 294

ggtacttctatcctgtctacctggattggcggtagcactcgttccatttccggtacgagc

G T S I **L** S T W I G G S T R S **I** S G T S 314

atggctactccacatgttgctggtctggcagcatacctgatgaccctgggtaagaccact

M A T P H V A G L A A Y L M T L G **K** T T 334

gctgcatccgcttgtcgttacatcgcggatactgcgaacaaaggcgatctgtctaacatc

A A **S** A C R Y I A D T A N K G D L S N I 354

ccgttcggcaccgttaatctgctggcatacaacaactatcaggctgtcgaccatcatcat

**P** F G T V N L L A Y N N Y Q A V D H H H 374

catcatcattga

H H H -

**Supporting Material Figure 1. DNA and amino acid sequences of the proteinase K wild type (WT)**

The DNA sequence is shown in lower case. The corresponding amino acid sequence is shown below in upper case. Positions at which amino acid substitutions were made are highlighted in bold and underlined. The substitutions are N95C, P97S, S107D, S123A, I132V, E138A, M145F, Y151A, V167I, L180I, Y194S, A199S, K208H, A236V, R237N, P265S, V267I, S273T, G293A, L299C, I310K, K332R, S337N and P355S (amino acids are numbered from the alanine following the NcoI site).

**Identification of genes homologous to proteinase K**

To select the substitutions, a set of serine proteases with >30% amino acid identity to proteinase K were identified using the proteinase K sequence to BLAST against Genbank. Fifty-nine sequences were identified, falling into 3 groups. Group A contained the wild type and 5 close homologs (>90% amino acid identity): GenBank accession numbers: gi|131077 (wt); gi|131084; gi|230675; gi|494434; gi|14278658 and gi|224977. Group B contained 42 more distant homologs (between 30% and 90% amino acid identity): gi|18542429; gi|4761119; gi|19171215; gi|16215662; gi|16215677; gi|117631; gi|6624958; gi|16215669; gi|742825; gi|628051; gi|16506136; gi|2351388; gi|14626933; gi|16506134; gi|6634475; gi|16506140; gi|8671180; gi|16215666; gi|16215664; gi|10181226; gi|28918475; gi|460032; gi|639712; gi|16215671; gi|7543916; gi|19171217; gi|19171219; gi|19171221; gi|9971109; gi|131088; gi|4092486; gi|56160990; gi|114081; gi|24528136; gi|15808791; gi|22652141; gi|15808805; gi|15640187; gi|5813790; gi|23894244 and gi|24528132. Group C contained 11 homologs (>30% amino acid identity) that were either reported in the literature to be thermostable or were >90% identical to a known thermostable sequence: gi|224461; gi|224450; gi|230344; gi|230758; gi|230927; gi|135738; gi|67623; gi|494465; gi|1711343; gi|30020448 and gi|29895999.

**Mapping selected substitution positions onto the proteinase K structure**

The proteinase K structure has been solved to 2.2Ǻ with a hexapeptide inhibitor (ref# 1PEK in the Protein Data Bank [5]. We mapped all of the substitution positions onto this structure. The mapping can be viewed dynamically in Supporting Material Figure 2, or as two separate static views in Supporting Material Figures 3 and 4.

**Supporting Material Figure 2 [see Additional file 3]. Positions of amino acid substitutions mapped onto the structure of proteinase K (pdb file)**

Positions where substitutions were beneficial (S123, I132, L180, K208, V267, S273, K332 and S337) are colored pink; positions where substitutions were strongly beneficial (Y151 and G293) are colored orange; positions where substitutions were detrimental (N95, P97, S107, E138, M145, V167, Y194, A199, A236, R237, P265, L299, I310 and P355) are colored green. Numbering is offset by 90 amino acids from the solved structure, so N95 appears as N5 in the figure. The image should be opened using Swiss Protein Data Bank Viewer [6]. The position of the substrate analog is shown in red.

**Supporting Material Figure 3 [see Additional file 4]. Positions of amino acid substitutions mapped onto the structure of proteinase K (Powerpoint file)**

Positions where substitutions were beneficial (S123, I132, L180, K208, V267, S273, K332 and S337) are colored pink; positions where substitutions were strongly beneficial (Y151 and G293) are colored orange; positions where substitutions were detrimental (N95, P97, S107, E138, M145, V167, Y194, A199, A236, R237, P265, L299, I310 and P355) are colored green. The position of the substrate analog is shown in red.

**Supporting Material Figure 4 [see Additional file 5]. Positions of amino acid substitutions mapped onto the structure of proteinase K (Powerpoint file)**

Positions where substitutions were beneficial (S123, I132, L180, K208, V267, S273, K332 and S337) are colored pink; positions where substitutions were strongly beneficial (Y151 and G293) are colored orange; positions where substitutions were detrimental (N95, P97, S107, E138, M145, V167, Y194, A199, A236, R237, P265, L299, I310 and P355) are colored green. The position of the substrate analog is shown in red.

# Machine learning analysis of variant sequences and activities

To analyze the effect of substitutions on proteinase K we first eliminated 5 of the 24 substitutions that appeared to destroy proteinase K activity: N95C, E138A, A236V, P97S and L299C (All 29 variants that contained any of theses 5 substitutions had zero activity and were removed from the analysis). We then used 8 different machine learning algorithms to minimize the discrepancy between measured activity y­_i_ and the activity predicted by the weight vector **w**, calculated as ỹ_i_=(∑_j=1..19_w_j_x_i,j_)_+_w_20_. The minimization problem has the following form

minimize **_w_** ( ∑_i_ *Loss*(y­_i_,ỹ_i_) + C *Reg*(**w**) ),

where Loss(y­_i_,ỹ_i_) is a non-negative measure of discrepancy between the activity y­_i_ and its estimate ỹ_i_, Reg(**w**) is a non-negative regularization function for weight vector **w**, and C is a non-negative constant that specifies a trade-off between these two functions.

We wanted to start simple and therefore chose to model our activity as a linear combination of 19 features (1 for each of the 19 remaining amino acid substitutions) and a constant offset. Later we also added a small number of interactive terms, i.e. quadratic features of the form x_i_ x_j_ where 1 <=i, j<=20. We chose regression over classification models because we wanted to make optimal use of the activities rather than throwing away information by thresholding the feature values that are so expensive to come by.

The goal of each algorithm was to achieve accurate linear estimates of the activities of the training examples. However the weight vectors produced by the algorithms vary because they are designed to optimize different regularizations and loss functions as shown in Supporting Material Table 1. The PLSR minimization uses square loss, but the presentation would be too lengthy here [7].

**Supporting Material Table 1.** Regularization and loss functions for analysis methods used.

|  | $Loss(y_{i},\tilde{y}_{i})$ | *Reg*(**w**) |
| --- | --- | --- |
| **RR** | square loss: $(y_{i}-\tilde{y}_{i})^{2}$ | two-norm : $\sum_{j=1..19} w_{j}^{2}$ |
| **Lasso** | square loss | one-norm :$\sum_{j=1..19} \left\vert w_{j}\right\vert$ |
| **SVMR** | ε-sensitive absolute loss: $\left\{\begin{matrix} \left\vert y_{i}-\tilde{y}_{i}\right\vert\text{ if }\left\vert y_{i}-\tilde{y}_{i}\right\vert\text{ }\geq\varepsilon\\ \text{0 if }\left\vert y_{i}-\tilde{y}_{i}\right\vert\text{ }<\varepsilon\end{matrix}\right.$ | two-norm |
| **LPSVMR** | ε-sensitive absolute loss | one-norm |
| **LPBoostR** | ε-sensitive absolute loss | ε  and constraint $\sum_{j=1..20} \left\vert w_{j}\right\vert=1$ |
| **MR** | matching loss:  where $h(x)=1/(1+e^{-(\frac{x-a}{b})})$ is the transfer function. | two-norm |
| **ORMR** | matching loss | one-norm |

The trade-off coefficient C in above algorithms and the parameter ε in SVMR and LPSVMR were tuned with cross-validation. For the matching loss function, parameter *a* was set to be slightly larger than the maximum activity in the training set and parameter *b* was chosen from experience. All algorithms were run using a commercially available MattLab package from Mathworks [8].

**SUPPORTING MATERIAL REFERENCES**
