# Supplementary material for: Engineering proteinase K using machine learning and synthetic genes
Source: BMC Biotechnol. 2007 Mar 26;7:16. doi: 10.1186/1472-6750-7-16 (PMC1847811; doi:10.1186/1472-6750-7-16)
Supplement: Additional File 2 — Table 2. Contains sequence and activity information for all variants tested in this study. [file 1472-6750-7-16-S2.doc]

**Table 2. Sequences, activities and design methods for the 95 proteinase K variants used in this study.**

| Design  method | Variant | 95 | 97 | 107 | 123 | 132 | 138 | 145 | 151 | 167 | 180 | 194 | 199 | 208 | 236 | 237 | 265 | 267 | 273 | 293 | 299 | 310 | 332 | 337 | 355 | Activity  (+ heat) | | Activity (no heat) | |
| --- | --- | --- | --- | --- | --- | --- | --- | --- | --- | --- | --- | --- | --- | --- | --- | --- | --- | --- | --- | --- | --- | --- | --- | --- | --- | --- | --- | --- | --- |
|  | wt | N | P | S | S | I | E | M | Y | V | L | Y | A | K | A | R | P | V | S | G | L | I | K | S | P | 0.7 | 1.3 | 1.1 | 0.9 |
| A | 1-1 |  |  |  |  |  |  |  |  |  |  | S |  |  |  |  |  |  |  |  |  |  |  |  |  | 0 |  | 0.03 | 0.02 |
| B | 1-2 |  |  |  | A |  |  |  | A |  |  |  |  |  |  |  |  |  |  | A |  | K | R |  | S | 0.04 |  | 0.3 | 0.2 |
| B | 1-3 | C |  |  |  |  |  | F |  | I |  |  | S |  |  | N |  |  | T |  |  |  |  |  |  | 0 |  |  | 0 |
| B | 1-4 |  | S |  |  |  | A |  |  |  | I | S |  |  | V |  |  | I |  |  |  |  |  |  |  | 0 |  |  | 0 |
| B | 1-5 |  |  | D |  | V |  |  |  |  |  |  |  | H |  |  | S |  |  |  | C |  |  | N |  | 0 |  |  | 0 |
| B | 1-6 |  |  |  |  | V |  | F | A | I |  |  |  |  |  |  |  |  | T |  |  |  |  | N |  | 0.03 |  | 0.01 | 0.01 |
| B | 1-7 |  | S | D |  |  |  |  |  |  | I |  |  |  | V | N |  |  |  |  |  | K |  |  |  | 0 |  |  | 0 |
| B | 1-8 |  |  |  | A |  | A |  |  |  |  |  | S | H |  |  | S |  |  |  |  |  |  |  | S | 0 |  |  | 0 |
| B | 1-9 | C |  |  |  |  |  |  |  |  |  | S |  |  |  |  |  | I |  | A | C |  | R |  |  | 0 |  |  | 0 |
| B | 1-10 | C |  |  |  | V | A | F |  | I |  |  |  | H |  |  |  |  |  |  |  |  |  |  |  | 0 |  |  | 0 |
| B | 1-11 |  | S |  | A |  |  |  |  |  |  |  |  |  |  |  | S |  |  |  | C | K |  | N |  | 0 |  |  | 0 |
| B | 1-12 |  |  | D |  |  |  |  | A |  | I | S | S |  |  |  |  | I |  |  |  |  |  |  |  | 0.04 |  | 0.03 | 0.01 |
| B | 1-13 |  |  |  |  |  |  |  |  |  |  |  |  |  |  | N |  |  | T | A |  |  | R |  | S | 0.7 | 0.02 | 1.7 | 1.2 |
| B | 1-14 | C |  | D | A |  |  |  |  |  | I | S |  |  |  |  |  |  |  |  |  |  |  | N |  | 0 |  |  | 0 |
| B | 1-15 |  |  |  |  |  | A |  | A | I |  |  | S | H |  |  |  |  |  |  | C |  |  |  |  | 0 |  |  | 0 |
| B | 1-16 |  | S |  |  |  |  | F |  |  |  |  |  |  |  | N |  |  | T | A |  | K |  |  |  | 0 |  |  | 0 |
| B | 1-17 |  |  |  |  | V |  |  |  |  |  |  |  |  | V |  | S | I |  |  |  |  | R |  | S | 0 |  |  | 0 |
| B | 1-18 |  | S |  |  |  |  |  | A |  |  |  | S |  | V |  |  |  |  |  | C |  |  |  | S | 0 |  |  | 0 |
| B | 1-19 | C |  | D |  |  |  |  |  | I | I |  |  |  |  |  |  |  |  | A |  | K |  |  |  | 0 |  |  | 0 |
| B | 1-20 |  |  |  |  |  |  | F |  |  |  |  |  |  |  | N | S | I |  |  |  |  | R | N |  | 0 |  |  | 0 |
| B | 1-21 |  |  |  | A | V | A |  |  |  |  | S |  | H |  |  |  |  | T |  |  |  |  |  |  | 0 |  |  | 0 |
| B | 1-22 |  |  |  | A |  |  |  |  |  |  |  |  | H | V |  |  | I |  | A | C |  |  |  |  | 0 |  |  | 0 |
| B | 1-23 |  |  | D |  | V | A | F |  |  |  |  |  |  |  |  |  |  |  |  |  |  |  | N | S | 0 |  |  | 0 |
| B | 1-24 |  | S |  |  |  |  |  |  |  | I | S | S |  |  |  | S |  |  |  |  | K |  |  |  | 0 |  |  | 0 |
| B | 1-25 | C |  |  |  |  |  |  | A | I |  |  |  |  |  | N |  |  | T |  |  |  | R |  |  | 0 |  |  | 0 |
| C | 1-26 | C |  |  |  |  |  |  |  |  |  |  |  |  |  |  |  |  |  |  |  |  |  |  |  | 0 |  |  | 0 |
| C | 1-27 |  |  |  |  |  | A |  |  |  |  |  |  |  |  |  |  |  |  |  |  |  |  |  |  | 0 |  |  | 0 |
| C | 1-28 |  |  |  |  |  |  |  |  |  |  |  |  | H |  |  |  |  |  |  |  |  |  |  |  | 0.7 | 0.9 | 0.9 | 1.0 |
| C | 1-29 |  |  |  |  |  |  |  |  |  |  |  |  |  | V |  |  |  |  |  |  |  |  |  |  | 0 |  | 0 |  |
| C | 1-30 |  |  |  |  |  |  |  |  |  |  |  |  |  |  | N |  |  |  |  |  |  |  |  |  | 0.5 | 0.5 | 1.2 | 1.3 |
| C | 1-31 |  |  |  |  |  |  |  |  |  |  |  |  |  |  |  | S |  |  |  |  |  |  |  |  | 0.06 |  | 0.3 | 0.2 |
| C | 1-32 |  | S |  |  |  |  |  |  |  |  |  |  |  |  |  |  |  |  |  | C |  |  |  |  | 0 |  |  | 0 |
| D | 1-33 |  | S |  |  |  |  |  |  |  |  | S |  |  |  |  |  |  |  |  | C |  |  |  |  | 0 |  |  | 0 |
| D | 1-34 |  |  | D | A |  |  | F |  |  |  |  |  |  |  |  |  |  |  |  |  |  |  |  |  | 0 |  |  | 0 |
| D | 1-35 |  |  |  |  |  |  |  |  |  |  | S | S |  |  |  |  | I |  |  |  |  |  |  |  | 0.02 | 0.05 |  | 0.01 |
| D | 1-36 |  |  |  |  |  |  |  |  |  |  |  |  |  |  |  |  |  | T | A |  | K |  |  |  | 0 |  | 0.4 | 0.5 |
| D | 1-37 |  |  |  |  |  |  |  |  |  |  |  |  |  |  |  |  |  |  |  |  |  | R | N | S | 0 |  | 1.4 | 1.2 |
| D | 1-38 |  |  |  |  |  |  | F |  | I |  | S |  |  |  |  |  |  |  |  |  |  |  |  |  | 0 |  | 0.01 | 0.04 |
| D | 1-39 |  |  |  |  |  |  |  |  |  | I |  | S |  |  |  |  |  | T |  |  |  |  |  |  | 0 |  | 0.2 | 0.5 |
| D | 1-40 |  |  |  |  |  |  |  |  |  |  |  |  |  |  |  |  | I |  | A |  |  | R |  |  | 2.6 | 3.8 | 1.7 | 1.3 |
| D | 1-41 |  |  | D |  |  |  |  |  |  |  |  |  |  |  |  |  |  |  |  |  | K |  | N |  | 0 |  | 0.05 | 0.1 |
| D | 1-42 |  |  |  | A |  |  |  | A |  |  |  |  |  |  |  |  |  |  |  |  |  |  |  | S | 0 |  | 0.4 | 0.6 |
| D | 1-43 |  |  |  |  |  |  |  | A | I | I |  |  |  |  |  |  |  |  |  |  |  |  |  |  | 0.8 | 0.2 | 1.3 | 1.8 |
| E | 1-44 |  |  | D |  |  |  |  | A |  |  | S |  |  |  |  |  |  | T |  |  |  | R |  |  | 0 |  | 0.01 | 0.03 |
| E | 1-45 |  |  |  | A |  |  |  |  | I |  |  | S |  |  |  |  |  |  | A |  |  |  | N |  | 0 |  | 0.7 | 1.0 |
| E | 1-46 |  |  |  |  |  |  | F |  |  | I |  |  |  |  |  |  | I |  |  |  | K |  |  | S | 0 |  | 0.4 | 0.5 |
| E | 1-47 |  |  | D |  |  |  |  |  | I |  |  |  |  |  |  |  | I | T |  |  |  |  | N |  | 0 |  | 1.1 | 1.3 |
| E | 1-48 |  |  |  | A |  |  |  |  |  | I | S |  |  |  |  |  |  |  | A |  |  |  |  | S | 0 |  | 0.04 | 0.1 |
| E | 1-49 |  |  |  |  |  |  | F | A |  |  |  | S |  |  |  |  |  |  |  |  | K | R |  |  | 0 |  |  | 0 |
| F | 1-50 |  |  |  |  |  |  |  |  |  |  |  |  | H |  |  |  | I |  | A |  |  | R |  |  | 3.7 | 3.4 | 1.1 | 1.3 |
| F | 1-51 |  |  |  |  |  |  |  |  | I |  |  |  |  |  |  |  | I |  | A |  |  | R | N |  | 0.43 |  |  | 0.9 |
| F | 1-52 |  |  |  |  |  |  |  |  |  |  |  |  |  |  | N |  | I |  | A |  |  | R |  | S | 0.02 | 0.02 |  | 0.5 |
| F | 1-53 |  |  |  |  |  |  |  |  | I |  |  |  |  |  | N |  |  |  | A |  |  | R |  | S | 0 |  |  | 0.8 |
| F | 1-54 |  |  |  |  |  |  |  |  | I |  |  |  |  |  | N |  | I |  | A |  |  | R | N | S | 0.07 |  |  | 0.9 |
| F | 1-55 |  |  |  |  |  |  |  |  | I |  |  |  |  |  |  |  | I |  | A |  |  | R |  |  | 0.1 | 0.5 |  | 0.3 |
| F | 1-56 |  |  |  |  |  |  |  |  |  |  |  |  |  |  |  |  | I |  |  |  |  | R | N | S | 0 |  |  | 0.3 |
| F | 1-57 |  |  |  |  |  |  |  |  | I |  |  |  |  |  |  |  | I | T |  |  |  | R | N |  | 0.07 | 0.08 |  | 0.2 |
| F | 1-58 |  |  |  |  |  |  |  |  | I |  |  |  | H |  | N |  | I |  | A |  |  | R | N | S | 0.04^1^ | 0.05^2^ |  | 0.4 |
| F | 1-59 |  |  |  |  |  |  |  |  | I |  |  |  | H |  |  |  | I |  | A |  |  | R |  |  | 0.2^3^ | 0.8^3^ | 0.7 | 0.2 |
|  |  |  |  |  |  |  |  |  |  |  |  |  |  |  |  |  |  |  |  |  |  |  |  |  |  |  |  |  |  |
| G | 2-1 |  |  |  |  |  |  |  |  |  | I |  |  | H |  |  |  | I |  | A |  |  | R |  |  | 3.5 | 1.9 |  |  |
| G | 2-2 |  |  |  |  |  |  |  |  |  |  |  |  | H |  |  |  | I |  | A |  |  |  |  |  | 4.0 | 2.3 |  |  |
| G | 2-3 |  |  |  |  |  |  |  |  |  |  |  |  | H |  |  |  |  |  | A |  |  |  |  |  | 5.5 | 3.2 |  |  |
| G | 2-4 |  |  |  |  |  |  |  |  |  | I |  |  | H |  |  |  |  |  | A |  |  | R |  |  | 3.6 | 6.0 |  |  |
| G | 2-5 |  |  |  |  |  |  |  | A |  | I |  |  | H |  |  |  | I |  | A |  |  | R |  |  | 3.9 | 4.7 |  |  |
| G | 2-6 |  |  |  |  |  |  |  |  |  |  |  |  | H |  |  |  |  |  | A |  |  | R |  |  | 4.0 | 3.8 |  |  |
| H | 2-7 |  |  |  |  | V |  |  | A |  |  |  |  |  |  |  |  | I |  | A |  |  | R |  |  | 6.4 | 6.7 |  |  |
| H | 2-8 |  |  |  |  | V |  |  |  |  |  |  | S | H |  |  |  | I |  | A |  |  | R |  |  | 0 | 0 |  |  |
| H | 2-9 |  |  |  | A |  |  | F |  |  |  |  |  | H |  |  |  | I |  | A |  |  | R |  |  | 0.1 | 0.3 |  |  |
| H | 2-10 |  |  |  |  | V |  | F |  |  | I |  |  |  |  |  |  | I |  | A |  |  | R |  |  | 0.3 | 0.1 |  |  |
| H | 2-11 |  |  |  | A |  |  | F |  |  | I |  |  | H |  |  |  |  |  | A |  |  |  |  |  | 0.1 | 0.1 |  |  |
| H | 2-12 |  |  | D |  |  |  |  | A |  | I |  |  | H |  |  |  |  |  | A |  |  |  |  |  | 3.0 | 6.8 |  |  |
| I | 2-13 |  |  |  |  |  |  |  |  | I | I |  |  |  |  |  |  | I |  | A |  |  | R |  |  | 2.2 | 1.4 |  |  |
| I | 2-14 |  |  |  |  |  |  |  |  |  |  |  |  |  |  |  |  |  |  | A |  |  |  |  |  | 4.2 | 5.1 |  |  |
| I | 2-15 |  |  |  |  |  |  |  |  |  |  |  |  |  |  |  |  |  |  |  |  |  | R |  |  | 0.8 | 1.3 |  |  |
| I | 2-16 |  |  |  |  |  |  |  |  |  |  |  |  |  |  |  |  |  |  | A |  |  | R |  |  | 4.9 | 3.9 |  |  |
| I | 2-17 |  |  |  |  |  |  |  |  | I |  |  |  |  |  |  |  | I |  |  |  |  |  |  |  | 1.0 | 0.9 |  |  |
| I | 2-18 |  |  | D |  |  |  |  |  |  | I |  |  |  |  |  |  | I |  |  |  |  | R |  |  | 0.4 | 0.3 |  |  |
| I | 2-19 |  |  |  |  |  |  |  |  | I |  |  |  | H |  |  |  | I | T | A |  |  | R |  |  | 2.8 | 2.8 |  |  |
| I | 2-20 |  |  | D |  |  |  |  | A |  |  |  |  |  |  |  |  | I |  | A |  |  |  |  |  | 3.6 | 3.2 |  |  |
|  |  |  |  |  |  |  |  |  |  |  |  |  |  |  |  |  |  |  |  |  |  |  |  |  |  |  |  |  |  |
| J | 3-1 |  |  |  |  | V |  |  | A |  |  |  |  | H |  |  |  |  | T | A |  |  | R |  |  | 3.2 |  |  |  |
| J | 3-2 |  |  |  |  | V |  |  | A |  |  |  |  | H |  |  |  |  | T | A |  |  | R | N |  | 5.9 | 5.5 |  |  |
| J | 3-3 |  |  |  |  |  |  |  | A |  |  |  |  | H |  |  |  |  |  | A |  |  | R | N |  | 9.8 |  |  |  |
| J | 3-4 |  |  |  | A | V |  |  | A |  | I |  |  | H |  |  |  |  | T | A |  |  |  | N |  | 6.8 |  |  |  |
| J | 3-5 |  |  |  |  | V |  |  | A |  |  |  |  | H |  |  |  |  | T | A |  |  |  | N |  | 7.5 | 7.1 |  |  |
| K | 3-6 |  |  |  |  |  |  |  | A |  |  |  |  | H |  |  |  |  |  | A |  |  |  |  |  | 16.8 | 16.2 |  |  |
| K | 3-7 |  |  |  |  |  |  |  | A |  |  |  |  | H |  |  |  |  | T | A |  |  |  |  |  | 7.0 | 6.3 |  |  |
| K | 3-8 |  |  |  |  |  |  |  | A |  |  |  |  |  |  |  |  |  |  | A |  |  |  |  |  | 9.1 | 17.5 |  |  |
| K | 3-9 |  |  |  |  | V |  |  | A |  | I |  |  |  |  |  |  |  | T | A |  |  |  |  |  | 10.7 | 8.4 |  |  |
| L | 3-10 |  |  |  |  | V |  |  | A |  | I |  |  |  |  |  |  | I | T | A |  |  |  |  |  | 10.2 | 7.2 |  |  |
| L | 3-11 |  |  |  |  |  |  |  | A |  |  |  |  | H |  |  |  |  | T | A |  |  | R | N |  | 24.9 | 15.7 |  |  |
| L | 3-12 |  |  |  |  |  |  |  | A |  | I |  |  | H |  |  |  |  | T | A |  |  | R | N |  | 37.4 | 12.2 |  |  |
| L | 3-13 |  |  |  |  |  |  |  | A |  |  |  |  | H |  |  |  |  |  | A |  |  | R |  |  | 17.0 | 8.8 |  |  |
| L | 3-14 |  |  |  | A |  |  |  | A |  | I |  |  | H |  |  |  |  | T | A |  |  |  |  |  | 5.5 |  |  |  |
| L | 3-15 |  |  |  |  |  |  |  | A |  |  |  |  |  |  |  |  |  | T | A |  |  |  |  |  | 14.4 | 8.1 |  |  |
| L | 3-16 |  |  |  |  |  |  |  | A |  |  |  |  |  |  |  |  | I | T | A |  |  | R |  |  | 8.5 | 5.6 |  |  |

The header shows the 24 substitution positions (See Additional file 1 for the positions of these within the sequence). The amino acids sequence of the wild type (WT) is shown in the first row. The substitutions from WT are indicated at each position for each variant n-m, where n indicates the set number and m the variant number within the set.

The right hand columns show the activity measured for each variant, as described in the Methods section. Proteinase K variants were expressed in *E. coli*, purified on Ni-NTA and eluted with imidazole. Proteins were diluted directly ("No Heat", far right columns) or following a 5 minute heating step at 68°C ("+ Heat" columns) into reaction buffer containing 500 μM substrate (N-Succinyl-Ala-Ala-Pro-Leu p-nitroanilide) at 37°C. Reactions were followed by measuring absorbance at 405 nm at 10 minute intervals. The initial rates were calculated by measuring the slopes of the curves, and are expressed relative to the mean activity of 2 replicates of the wild-type that was expressed and tested in the same experiment. Two variant sequences contained additional non-designed changes: 1-58 for which 2 different sequences were obtained with either ^1^K48S or ^2^T63S and 1-60 which contained one additional change ^3^A369V.

The method used to design each variant is indicated in the left hand column. Round 1 variants (see section 2.3): (A) random mutation; (B) random combinations of 6 of the initial 24 substitutions; (C) variants designed to test amino acid substitutions that did not appear in active variants from 1-2 to 1-25; (D) random combinations of 3 changes selected from the 19 substitutions after elimination of 5 "killer" changes; (E) random combinations of 5 changes selected from the 19 substitutions after elimination of 5 "killer" changes; (F) attempt to combine beneficial changes identified "by eye". Round 2 variants (see section 2.6): (G) machine learning design of "optimal" sequences using heated activity data; (H) machine learning design of "exploring" sequences using heated activity data; (I) machine learning design of "exploring" sequences using unheated activity data. Round 3 variants (see section 2.7): (J) machine learning designs using mean weights > 0; (K) machine learning designs using mean weights – standard deviation > 0; (L) machine learning designs using interactive terms.
