# Supplementary figures and images for: Engineering proteinase K using machine learning and synthetic genes
Source: BMC Biotechnol. 2007 Mar 26;7:16. doi: 10.1186/1472-6750-7-16 (PMC1847811; doi:10.1186/1472-6750-7-16)

## Slide 1
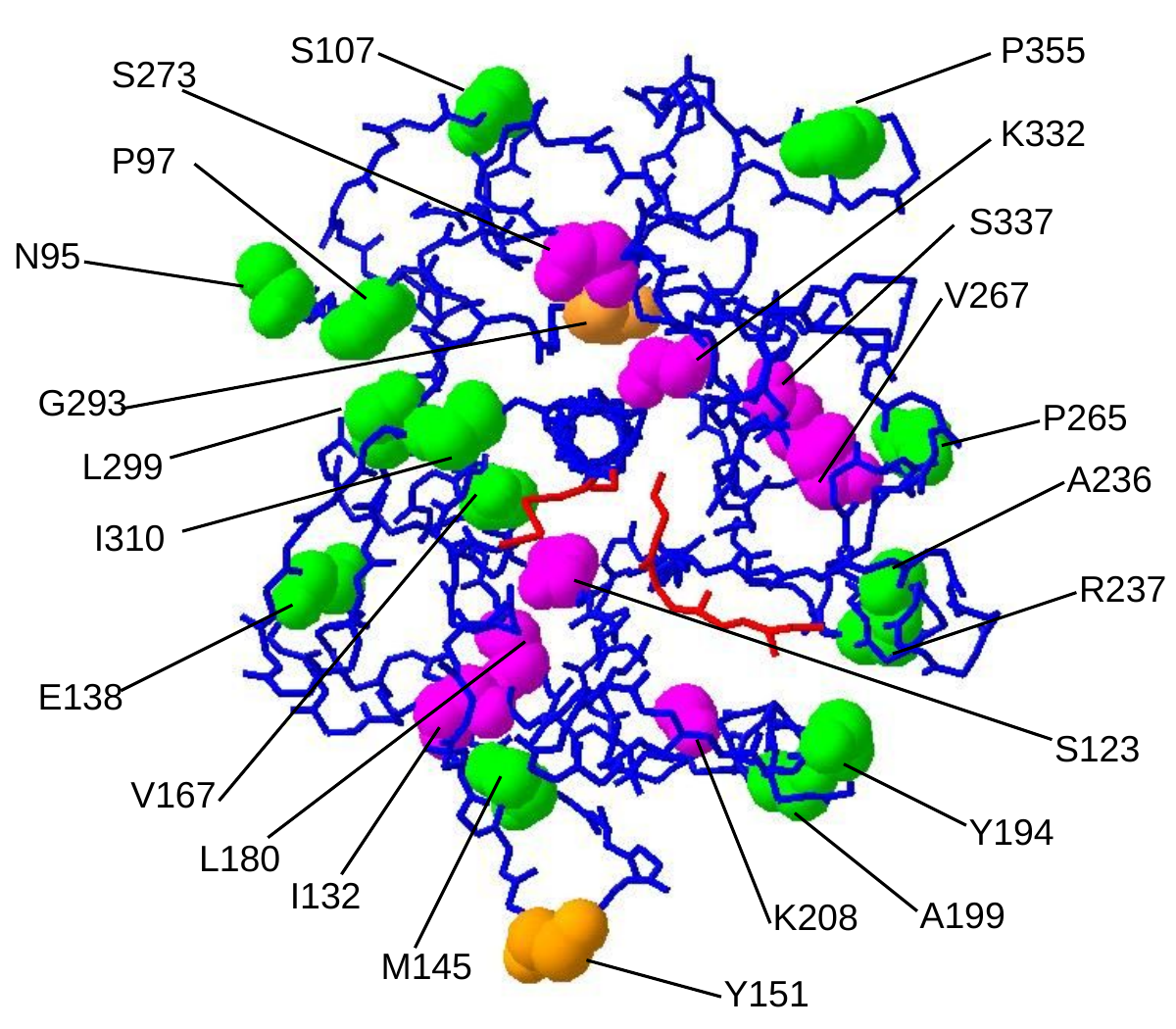

S107
P355
S273
K332
P97
S337
N95
V267
G293
P265
L299
A236
I310
R237
E138
S123
V167
Y194
L180
I132
A199
K208
M145
Y151

Supplement: Additional File 4 — Supporting Material Figure 3. Positions of amino acid substitutions mapped onto the structure of proteinase K. [file 1472-6750-7-16-S4.ppt]

## Slide 1
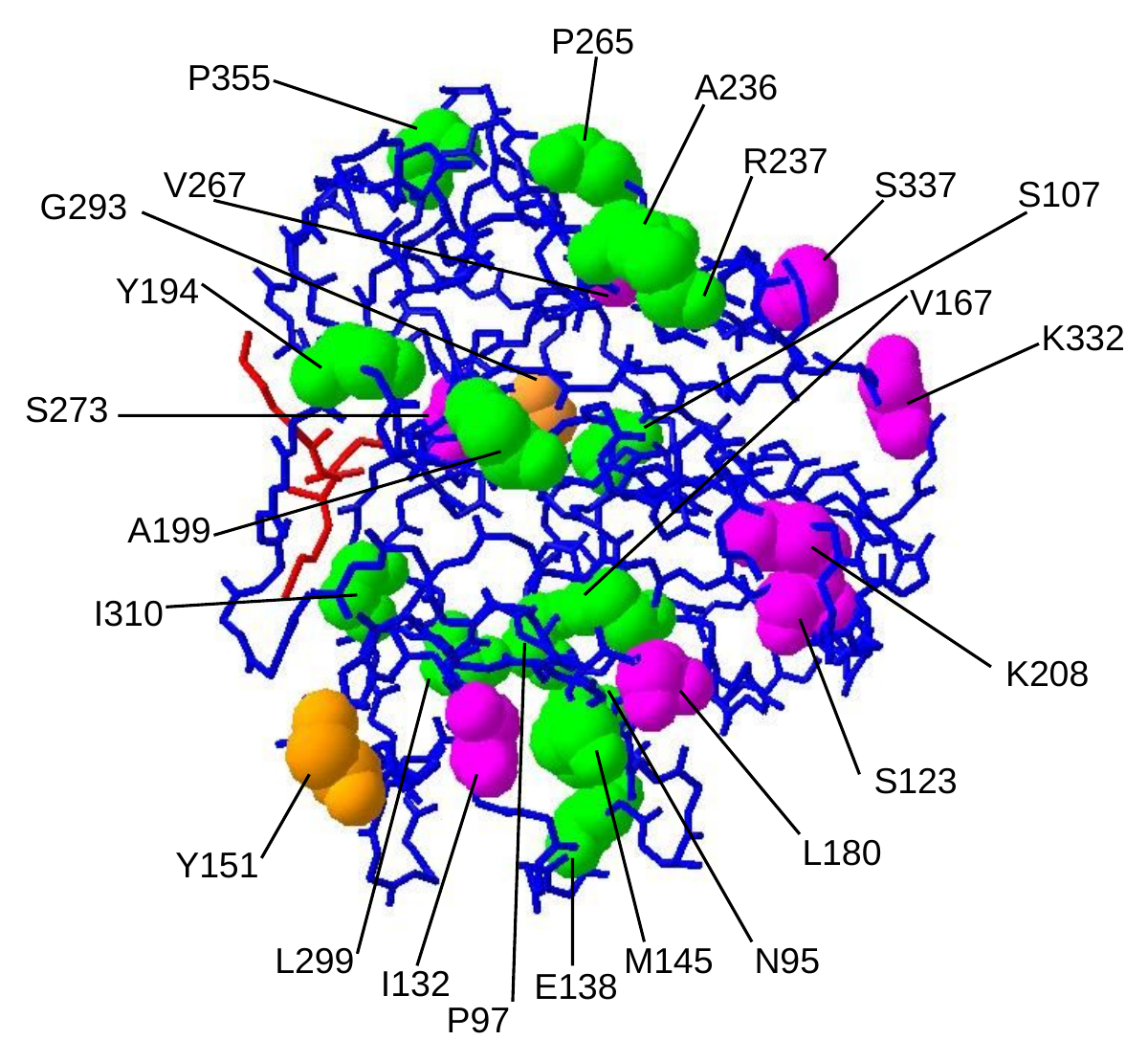

P265
P355
A236
R237
V267
S337
S107
G293
Y194
V167
K332
S273
A199
I310
K208
S123
L180
Y151
L299
M145
N95
I132
E138
P97

Supplement: Additional File 5 — Supporting Material Figure 4. Positions of amino acid substitutions mapped onto the structure of proteinase K. [file 1472-6750-7-16-S5.ppt]
